# Supplementary material for: A comparative analysis of soil physicochemical properties and microbial community structure among four shelterbelt species in the northeast China plain
Source: Microbiol Spectr. 2024 Feb 20;12(4):e03683-23. doi: 10.1128/spectrum.03683-23 (PMC10986494; doi:10.1128/spectrum.03683-23)
Supplement: Tables S1 to S5 — Bioinformatics processing data. [file spectrum.03683-23-s0001.docx]

Table S1. Summary of bacterial and fungal metabarcoding sequencing data.

|  | Bacteria | | | Fungi | | |
| --- | --- | --- | --- | --- | --- | --- |
| Sample ID | Clean Tags | Q 30% | OTU Number | Clean Tags | Q 30% | ASV Number |
| Jm.1 | 119170 | 93.6 | 4412 | 77929 | 99.2 | 362 |
| Jm.2 | 115181 | 93.5 | 4352 | 76118 | 99 | 493 |
| Jm.3 | 111116 | 93.7 | 4089 | 76655 | 98.9 | 420 |
| Fm.1 | 119891 | 93.5 | 4094 | 79439 | 99.1 | 497 |
| Fm.2 | 117638 | 93.6 | 4118 | 78428 | 99.1 | 495 |
| Fm.3 | 110070 | 93.3 | 4061 | 79110 | 99 | 498 |
| Am.1 | 113133 | 93.5 | 3246 | 83563 | 98.7 | 307 |
| Am.2 | 119553 | 93.3 | 4278 | 77517 | 98.8 | 444 |
| Am.3 | 109387 | 93.4 | 3896 | 77779 | 99.2 | 471 |
| Bp.1 | 113676 | 93.4 | 3554 | 78646 | 99.1 | 454 |
| Bp.2 | 119259 | 93 | 3712 | 81507 | 99.4 | 325 |
| Bp.3 | 117857 | 93.4 | 3406 | 79243 | 99.2 | 555 |

Table S2. The relative abundance in the phylum.

|  | Name | Jm | Fm | Am | Bp | Mean ± SD |
| --- | --- | --- | --- | --- | --- | --- |
| Bacterial | Acidobacteria | 20.81% | 25.31% | 26.68% | 29.92% | 25.68% ± 3.27% |
|  | Proteobacteria | 27.35% | 20.83% | 24.03% | 14.48% | 21.67% ± 4.75% |
|  | Actinobacteria | 16.21% | 13.89% | 11.54% | 12.37% | 13.50% ± 1.78% |
|  | Verrucomicrobia | 6.93% | 10.64% | 8.83% | 17.04% | 10.86% ± 3.80% |
|  | Bacteroidetes | 8.87% | 9.13% | 9.28% | 7.26% | 8.64% ± 0.81% |
|  | Chloroflexi | 7.92% | 6.04% | 5.99% | 4.14% | 6.02% ± 1.34% |
|  | Gemmatimonadetes | 4.76% | 5.32% | 5.53% | 6.80% | 5.60% ± 0.75% |
|  | Rokubacteria | 1.76% | 2.66% | 1.50% | 3.27% | 2.30% ± 0.71% |
|  | Planctomycetes | 1.12% | 1.20% | 1.11% | 0.97% | 1.10% ± 0.08% |
|  | Nitrospirae | 0.79% | 1.23% | 1.01% | 1.13% | 1.04% ± 0.16% |
|  | Patescibacteria | 1.05% | 0.78% | 1.38% | 0.52% | 0.93% ± 0.32% |
|  | Latescibacteria | 0.78% | 1.13% | 0.94% | 0.58% | 0.86% ± 0.20% |
|  | (Unassigned) | 0.44% | 0.51% | 0.49% | 0.36% | 0.45% ± 0.06% |
|  | Armatimonadetes | 0.34% | 0.47% | 0.56% | 0.28% | 0.41% ± 0.11% |
|  | Elusimicrobia | 0.24% | 0.27% | 0.43% | 0.24% | 0.30% ± 0.08% |
|  | others | 0.63% | 0.58% | 0.69% | 0.64% | 0.64% ± 0.04% |
| Fungi | Ascomycota | 55.50% | 62.37% | 66.77% | 73.07% | 64.43% ± 6.41% |
|  | Basidiomycota | 24.99% | 15.77% | 20.90% | 13.21% | 18.72% ± 4.56% |
|  | Mortierellomycota | 16.55% | 16.35% | 9.31% | 10.16% | 13.09% ± 3.37% |
|  | (Unassigned) | 2.53% | 4.72% | 2.71% | 2.60% | 3.14% ± 0.91% |
|  | Chytridiomycota | 0.17% | 0.39% | 0.05% | 0.64% | 0.31% ± 0.22% |
|  | Glomeromycota | 0.07% | 0.23% | 0.11% | 0.11% | 0.13% ± 0.06% |
|  | Olpidiomycota | 0.13% | 0.06% | 0.06% | 0.16% | 0.10% ± 0.05% |
|  | Cercozoa | 0.04% | 0.04% | 0.02% | 0.01% | 0.03% ± 0.01% |
|  | Mucoromycota | 0.02% | 0.02% | 0.00% | 0.03% | 0.02% ± 0.01% |
|  | Blastocladiomycota | 0.00% | 0.00% | 0.06% | 0.00% | 0.01% ± 0.03% |
|  | others | 0.00% | 0.04% | 0.00% | 0.00% | 0.01% ± 0.02% |

Table S3. The relative abundance in the genus.

|  | Name | Jm | Fm | Am | Bp | Mean ± SD |
| --- | --- | --- | --- | --- | --- | --- |
| Bacterial | *RB41* | 6.78% | 9.11% | 9.09% | 14.83% | 9.95% ± 2.97% |
|  | *Candidatus_Udaeobacter* | 4.48% | 7.91% | 5.64% | 15.20% | 8.31% ± 4.17% |
|  | *Candidatus_Solibacter* | 2.86% | 3.51% | 3.93% | 2.97% | 3.32% ± 0.43% |
|  | *Bryobacter* | 1.33% | 1.54% | 2.50% | 0.70% | 1.52% ± 0.65% |
|  | *Nitrospira* | 0.78% | 1.23% | 1.01% | 1.13% | 1.04% ± 0.17% |
|  | *Gemmatimonas* | 1.14% | 0.90% | 1.79% | 0.39% | 1.06% ± 0.50% |
|  | *Achromobacter* | 0.63% | 0.92% | 1.08% | 1.35% | 1.00% ± 0.26% |
|  | *Ellin6067* | 0.92% | 0.83% | 1.89% | 0.54% | 1.05% ± 0.51% |
|  | *Haliangium* | 1.18% | 1.17% | 1.12% | 0.51% | 1.00% ± 0.28% |
|  | *Acidothermus* | 0.81% | 1.03% | 0.56% | 1.29% | 0.92% ± 0.27% |
| Fungi | (Unassigned) | 16.82% | 22.32% | 16.02% | 16.32% | 17.97% ± 2.59% |
|  | *Mortierella* | 16.55% | 16.35% | 9.31% | 10.16% | 13.09% ± 3.37% |
|  | *Schizothecium* | 2.33% | 4.12% | 22.58% | 0.96% | 7.50% ± 8.78% |
|  | *Plectosphaerella* | 1.70% | 5.88% | 2.19% | 21.28% | 7.76% ± 7.97% |
|  | *Mrakia* | 13.81% | 2.47% | 4.43% | 7.79% | 7.12% ± 4.30% |
|  | *Didymella* | 9.35% | 3.81% | 3.98% | 6.23% | 5.84% ± 2.24% |
|  | *Fusarium* | 2.06% | 4.20% | 3.93% | 10.39% | 5.15% ± 3.14% |
|  | *Laetinaevia* | 6.18% | 7.36% | 2.64% | 1.96% | 4.53% ± 2.29% |
|  | *Waitea* | 5.12% | 0.02% | 8.14% | 0.02% | 3.32% ± 3.47% |
|  | *Lasiosphaeris* | 8.71% | 1.58% | 0.02% | 1.47% | 2.94% ± 3.38% |

Table S4. Variability of bacterial biomarkers in abundance between groups.

| Name | Group | LDA score | P-value |
| --- | --- | --- | --- |
| p_Proteobacteria | Jm | 4.88 | 0.050 |
| c_Alphaproteobacteria | Jm | 4.64 | 0.033 |
| g_*Pseudomonas* | Jm | 3.96 | 0.022 |
| 0_Pseudomonadales | Jm | 3.95 | 0.022 |
| f_Pseudomonadaceae | Jm | 3.93 | 0.022 |
| 0_Sphingomonadales | Jm | 3.93 | 0.031 |
| f_Roseiflexaceae | Jm | 3.92 | 0.050 |
| f_Sphingomonadaceae | Jm | 3.91 | 0.031 |
| 0_Chloroflexales | Jm | 3.89 | 0.050 |
| c_Chloroflexia | Jm | 3.89 | 0.050 |
| g_*Pseudarthrobacter* | Jm | 3.85 | 0.022 |
| 0_Micrococcales | Jm | 3.84 | 0.019 |
| f_Micrococcaceae | Jm | 3.84 | 0.022 |
| g_*Acidibacter* | Jm | 3.77 | 0.033 |
| 0_Gammaproteobacteria_Incertae_Sedis | Jm | 3.76 | 0.034 |
| g_*Niastella* | Jm | 3.74 | 0.044 |
| f_Micromonosporaceae | Jm | 3.73 | 0.049 |
| 0_Micromonosporales | Jm | 3.72 | 0.049 |
| 0_Microtrichales | Jm | 3.60 | 0.024 |
| c_Acidimicrobiia | Jm | 3.59 | 0.033 |
| f_Steroidobacteraceae | Jm | 3.56 | 0.019 |
| f_Ilumatobacteraceae | Jm | 3.56 | 0.033 |
| 0_Steroidobacterales | Jm | 3.56 | 0.019 |
| g_*Leptolyngbya_EcFYyyy* | Jm | 3.50 | 0.025 |
| c_Verrucomicrobiae | Bp | 4.93 | 0.033 |
| g_*Candidatus_Udaeobacter* | Bp | 4.92 | 0.033 |
| p_Verrucomicrobia | Bp | 4.91 | 0.033 |
| 0_Chthoniobacterales | Bp | 4.91 | 0.033 |
| f_Chthoniobacteraceae | Bp | 4.90 | 0.033 |
| g_*Methylomicrobium* | Bp | 3.69 | 0.040 |
| g_*Waddlia* | Bp | 3.63 | 0.013 |
| f_Waddliaceae | Bp | 3.61 | 0.013 |
| c_Gammaproteobacteria | Am | 4.59 | 0.034 |
| 0_Betaproteobacteriales | Am | 4.56 | 0.043 |
| 0_Solibacterales | Am | 4.43 | 0.041 |
| f_Solibacteraceae_Subgroup_3 | Am | 4.42 | 0.041 |
| c_Acidobacteriia | Am | 4.41 | 0.033 |
| f_Nitrosomonadaceae | Am | 4.30 | 0.034 |
| g_*Bryobacter* | Am | 4.19 | 0.024 |
| g_*Ellin6067* | Am | 4.09 | 0.045 |
| g_*Gemmatimonas* | Am | 4.08 | 0.022 |
| g_*Massilia* | Am | 3.96 | 0.041 |
| f_Micropepsaceae | Am | 3.84 | 0.024 |
| g_*Sphingomonas* | Am | 3.82 | 0.025 |
| 0_Micropepsales | Am | 3.82 | 0.024 |
| g_*MND1* | Am | 3.74 | 0.041 |

Table S5. Variability of fungi biomarkers in abundance between groups.

| Name | Group | LDA score | P-value |
| --- | --- | --- | --- |
| f_Pyronemataceae | Jm | 4.00 | 0.043 |
| g_*Volutella* | Jm | 3.60 | 0.047 |
| g_*Entoloma* | Jm | 3.12 | 0.024 |
| o_Agaricales | Fm | 4.06 | 0.038 |
| f_Bulleribasidiaceae | Fm | 4.01 | 0.043 |
| f_Clavicipitaceae | Fm | 3.89 | 0.033 |
| g_*Metarhizium* | Fm | 3.89 | 0.033 |
| g_*Vishniacozyma* | Fm | 3.82 | 0.027 |
| g_*Tylospora* | Fm | 3.74 | 0.038 |
| g_*Phialocephala* | Fm | 3.53 | 0.044 |
| f_Vibrisseaceae | Fm | 3.52 | 0.044 |
| g_*Clitopilus* | Fm | 3.51 | 0.023 |
| f_Entolomataceae | Fm | 3.39 | 0.027 |
| g_*Amanita* | Fm | 3.03 | 0.030 |
| o_Glomerellales | Bp | 5.08 | 0.024 |
| g_*Plectosphaerella* | Bp | 5.08 | 0.024 |
| f_Plectosphaerellaceae | Bp | 5.06 | 0.024 |
| f_Sordariales_fam_Incertae_sedis | Bp | 4.08 | 0.044 |
| g_*Ramophialophora* | Bp | 4.06 | 0.044 |
| g_*Leucoagaricus* | Bp | 3.50 | 0.042 |
| f_Agaricaceae | Bp | 3.49 | 0.042 |
| f_Inocybaceae | Bp | 3.14 | 0.034 |
| g_*Mallocybe* | Bp | 3.08 | 0.038 |
| g_*Schizothecium* | Am | 5.18 | 0.027 |
